# Accumulation of oocytes and/or embryos by vitrification: a new strategy for managing poor responder patients undergoing pre implantation diagnosis

**DOI:** 10.12688/f1000research.2-240.v2

**Published:** 2014-03-03

**Authors:** Alexia Chatziparasidou, Martine Nijs, Martha Moisidou, Oraiopoulou Chara, Christina Ioakeimidou, Christos Pappas, Nicos Christoforidis

**Affiliations:** 1 Embryolab, Assisted Reproduction Unit, Kalamaria, Thessaloniki, 551 34, Greece

## Abstract

**Background:** Low (or poor) responder patients are women who require large doses of stimulation medications and produce less than an optimal number of oocytes during IVF cycles. Low responder patients produce few oocytes and embryos, which significantly reduces their chances for success in a preimplantation genetic diagnosis (PGD) cycle. Accumulation of vitrified oocytes or embryos before the actual PGD cycle is a possible strategy that might increase patient’s chances for a healthy pregnancy.

**Aim of the study**: This retrospective study evaluates the efficacy of a PGD program in low responder patients after repeated ovarian stimulation cycles with cumulative vitrification of oocytes and embryos.

**Methods:** Over a period of 30 months, 13 patients entering the PGD program were identified as poor responders after their first ovarian stimulation. These patients started a PGD cycle for one of the following indications: history of recurrent implantation failure (n=1), cystic fibrosis (n=1), X-linked microtubular myopathy (n=1), recurrent miscarriages (n=5), Duchene muscular dystrophy (n=1), chromosomal translocation (n=1) and high sperm aneuploidy (n=1).  After multiple ovarian hormonal stimulations patients had either all mature oocytes (Group A; 3 patients) or all of their day 2 embryos vitrified (group B; 10 patients). Mean total number of oocyte collections per patient was 2.3 (range: 2 - 5 cycles).

**Results:** In the actual PGD cycle, all vitrified oocytes from group A patients were warmed and underwent intra cytoplasmic sperm injection (ICSI) followed by culture up to day 3. For group B patients all vitrified day 2 embryos were warmed and cultured overnight. On day 3 of culture, all embryos from Group A and B had blastomere biopsy followed by genetic analysis. In group A, 20 embryos were found suitable for biopsy and genetic analysis; at least one healthy embryo was available for transfer for each patient.  For group B, 72 embryos in total were available for biopsy and PGD.  All patients, except one, had at least one healthy day 5 embryo for transfer (mean number of 2.1 embryos per transfer). Nine patients had a clinical pregnancy; 7 patients delivered a healthy baby.

**Conclusion:** Low responder patients entering a PGD program might increase their chances for a healthy pregnancy by repeat ovarian stimulation in combination with cumulative oocyte or embryo vitrification.

## Introduction

Low responder patients undergoing hormonal stimulation for an IVF or ICSI treatment have a reduced potential to produce an adequate number of oocytes and hence also embryos ^1, 2^. Especially for patients seeking a healthy pregnancy through preimplantation genetic diagnosis (PGD), this low production of oocytes and embryo(s) in one cycle will significantly reduce their chances of success. Multiple consecutive ovarian stimulation cycles combined with serial vitrification of oocytes and embryos obtained before the actual PGD could be an option to increase the chances for these patients. Until now, only one successful case report has been presented by Chung *et al*. ^3^ where a normal birth was obtained after serial vitrification of oocytes from 5 consecutive ovarian stimulation cycles for a patient carrying reciprocal translocations.

This retrospective cohort study evaluates the efficacy of a PGD program in low responder patients after repeated ovarian stimulation and accumulation of vitrified oocytes or embryos before genetic analysis, in combination with PGD on embryos obtained from a fresh ICSI cycle.

## Methods

### Setting and study design

This retrospective cohort study was performed over a 30 month-period (2011–2013) at Embryolab, a private fertility treatment centre in Thessaloniki, Greece.

### Cycles and patients studied

During the 30 month period 13 patients of those entering the PGD program showed to be poor responders. Patients were counselled on both options (serial oocyte or embryo vitrification) with clear explanations on pro and cons of each option. Patients selected themselves for serial oocyte or embryo vitrification. PGD patients with more than 6 oocytes or 5 embryos from their first fresh PGD cycle were excluded from the study. Study patients started a PGD cycle for one of the following indications: history of recurrent implantation failure (n=1), cystic fibrosis (n=1), X-linked microtubular myopathy (n=1), recurrent miscarriages (n=5), Duchene muscular dystrophy (n=1), chromosomal translocation (n=1) and high sperm aneuploidy (n=1). Baseline characteristics for patients were mean age of 35,2 years; mean antral follicle count of 7; mean body mass index of 24,6 kg/m ^2^ and a mean FSH on day 2 of cycle: 7,43 IU.

### Ovarian stimulation of patients

Patient’s ovarian stimulation protocol consisted of a standard down-regulation protocol or antagonist protocol ^4^. Hormonal stimulation treatment showed these patients to be poor responders and very few oocytes could be harvested at the time of the first oocyte collection. Following counseling, couples opted for serial vitrification of oocytes (group A) or embryos (group B) from repeat ovarian stimulation cycles. Allocation to either group was based on the outcome of a medical counseling session with the patient. One to two extra hormonal stimulation cycles were initiated to obtain an accumulated minimum of 6 mature oocytes (group A) or alternatively of 5 embryos (group B) for each patient.

### IVF Laboratory protocols

Oocyte collection was carried out 36 hours post-hCG administration. Fresh semen samples were prepared by density gradient centrifugation and one wash step (Quinn's Advantage Sperm Washing Medium, Sage). ICSI was performed according to standard procedures ^5^. Oocytes were checked for presence of 2 pronuclei 18–22 hours post oocyte collection. Fertilised oocytes were group-cultured in 0.7 ml droplets (Cleavage medium, Sage) and embryo quality was checked daily under a microscope using a standard protocol ^10^. Oocytes and day 2 embryos were vitrified and warmed using the methods described by Kuwayama *et al*. (Cryotop, Cryotec, http://cryotech-japan.jp/method/warming_Protocol.htm) ^6^ and stored in liquid nitrogen.

### Pre implantation genetic diagnosis

Embryos were biopsied on day 3 of development. Three different genetic techniques were applied, depending on the indication: fluorescent *in situ* hybridization (FISH) ^7^ was used for patients suffering from X-linked microtubular myopathy, Duchene muscular dystrophy, high sperm aneuploidy or recurrent implantation failure; polymerase chain reaction (PCR) ^8^ was the technique used for patients at risk for offspring with cystic fibrosis. Array complete genome hybridisation techniques (aCGH) ^9^ were applied for patients at risk for recurrent miscarriage or for reciprocal translocations. Biopsied embryos were cultured individually in 50 μl droplets under oil (washed sterile oil, Sage, USA) until day 5 for transfer (Cleavage medium, Sage, USA). Embryo quality was checked daily under the microscope according to a standard protocol ^10^.

### Transfer of embryos

Embryo(s) were transferred under abdominal ultrasound guidance (Logic 400 MD) to the patient in 0.1 ml of medium (Cleavage medium, Sage) using a Wallace- (Smithsor Labotect soft catheter (Genetec). Clinical pregnancy was defined as the presence of a gestational sac with fetal heartbeat by ultrasound imaging at 8–10 weeks after embryo transfer.

### Laboratory quality

The IVF laboratory at Embryolab has ISO 9001:2000 accreditation (2007) and has been assessed in accordance to ISO 15189-2007.

Given the retrospective nature and lack of identifiable health data used in the study, no institutional review board approval was needed. Patients signed an informed consent before the start of the treatment.

## Results

During the 30 month study period, 13 patients were shown to be poor responders because of failure to produce a sufficient number of oocytes or embryos to continue their PGD analysis (< 6 mature oocytes or < 5 embryos on day 2). Mean age of the patients was 35.2 years (range: 31–41 years, SD: 3.4). After medical counseling all 13 patients agreed to accumulate their oocytes or embryos by vitrification, and hence underwent repeat hormonal stimulations and oocyte collections (mean: 2.3; range 2–5 stimulations) until a sufficient number was stored (>6 mature oocytes or >5 embryos on day 2). Mean total number of oocyte collections per patient (cycles) was 2.3 (range: 2–5 cycles). Details on laboratory and clinical outcomes are listed in Table 1. On day 3 of culture, a total of 92 embryos were biopsied and diagnosed genetically. In total 40 embryos were diagnosed as normal, 43 as abnormal and for 9 embryos no result was obtained. Mean number of biopsied embryos per patient was 7,2 (+SD: 2,1) and a mean average number of 2.1 embryos per patient were transferred. Eleven supernumerary embryos, diagnosed as being normal, were vitrified post-biopsy. One patient with a history of repeated failure of implantation had no healthy embryos available for transfer. This patient had a total of 5 embryos biopsied (2 from cryostorage and 3 fresh). Twelve out of 13 patients had an embryo transfer of a healthy embryo (92,3%) and 9 patients had a clinical pregnancy (75% clinical pregnancy rate in patients with embryo transfer). In total, 2 patients miscarried and 7 patients delivered a healthy baby (7/12; 58.3% delivery rate). Two twin pregnancies were noted; both patients had delivery of healthy babies. No frozen-thawed embryo transfer was done for any of the patients.

**Table 1.** Clinical and laboratory outcomes for poor responder PGD patients after serial vitrification of oocytes or embryos.

	Group A Vitrification of Oocytes	Group B Vitrification of Embryos
Number of patients with vitrification	3	10
Number of cycles with vitrification	6	18
Total number oocytes/embryos vitrified from repeat cycles	15	44
Survival after warming number (%)	15 (100%)	44 (100%)
Number of oocytes/embryos obtained in ultimate fresh cycle	22	28
Total number of embryos available for PGD on day 3	20	72
Number of patients with transfer of at least 1 healthy embryo	3	9
Mean number of embryos per transfer	2,1	
Number of patients with positive hCG test	9/12 (75.0%)	
Number of patients with healthy delivery	7/12 (58.3%)	

## Discussion

Low responder patients undergoing IVF are characterised by a low number of oocytes retrieved because of suboptimal oocyte maturation, poor embryo quality, hormonal stimulation cycle or embryo transfer cancellation ^2^. Cobo *et al*. ^11^ demonstrated in a prospective study that accumulation of oocytes by vitrification is a successful strategy for managing low responder patients in ‘classical’ IVF/ICSI treatments: delivery and cumulative delivery rates per patient were statistically higher in the low responder group (36.4%) than the low responder fresh group (23.7%). Our study could demonstrate, although on a limited number of patients, that this accumulation strategy can also be applied for a specific patient population, namely patients undergoing PGD for specific genetic diseases. Although we did not compare our outcomes to those of a control group of low responder fresh PGD patients from our center, we could demonstrate that the strategy to accumulate vitrified oocytes or embryos from consecutive hormonal stimulation cycles resulted in a sufficient number of embryos available for genetic diagnosis. As a consequence, a high percentage of patients had transfer of an embryo diagnosed to be negative for the specific genetic test (92,3%). It is evident that in order to accumulate oocytes and embryos by vitrification for the management of low responder patients, an efficient and well-established oocyte vitrification system needs to be in place. Survival rates after warming of these oocytes and embryos need to be optimal (between 80 and 100%); if this is not the case, this approach should not be offered to low responder patients. Our laboratory has high survival rates for oocytes and embryos (up to 100%) and comparable development and implantation rates for both fresh and vitrified embryos (Cryotop and Cryotec vitrification methods ^6, 11^) are obtained; hereby confirming outcomes of Rienzi *et al.* (2009) ^12^ and Ku *et al.* (2012) ^13^.

Although the treatment costs can be double or triple compared to one single hormonal stimulation for ICSI with PGD, the total costs of the accumulated cycles are lower because patients have to pay for only one ICSI procedure (in case of accumulation of oocytes) and only one genetic analysis combined with one embryo transfer.

Moreover, this accumulation strategy resulted in higher outcomes (58.3% delivery rate per transfer) as compared to the 24% delivery rate per fresh embryo transfer presented by the ESHRE PGD consortium for 2008 ^14^.

This retrospective cohort study demonstrates, although on a limited number of patients, that low responder patients in need of PGD can benefit from serial vitrification of oocytes and/or embryos after repeated ovarian stimulation cycles to improve their chances of a successful pregnancy. Future studies should address the ideal number of vitrified oocytes and/or embryos necessary in order to increase success in low responder patients undergoing PGD.
