# Supplementary material for: Accumulation of oocytes and/or embryos by vitrification: a new strategy for managing poor responder patients undergoing pre implantation diagnosis
Source: F1000Res. 2014 Mar 3;2:240. Originally published 2013 Nov 12. [Version 2] doi: 10.12688/f1000research.2-240.v2 (PMC4111112; doi:10.12688/f1000research.2-240.v2)
Supplement: Peer review history

# Peer review: Referee response for version 2

**DOI:** 10.5256/f1000research.3961.r3937

**Published:** 2014-07-03

**Authors:** Pedro Barri

**Affiliations:** 1 Department of Obstetrics, Institut Universitari Dexeus of the Universitat Autònoma de Barcelona, Barcelona, Spain

I have carefully read the new version of the article “Accumulation of oocytes and/or embryos by vitrification: a new strategy for managing poor responder patients undergoing pre implantation diagnosis”.

I thank the authors for having implemented the suggested changes. I feel comfortable with the article like it is now.

I have read this submission. I believe that I have an appropriate level of expertise to confirm that it is of an acceptable scientific standard.

# Peer review: Referee response for version 2

**DOI:** 10.5256/f1000research.3961.r4796

**Published:** 2014-05-19

**Authors:** Alex Simon

**Affiliations:** 1 Department of Obstetrics and Gynecology, Hadassah Medical Center, Jerusalem, Israel

In the current study the authors suggest to accumulate either embryos or oocytes for PGD/PGS in patients who are low responders. This method was found to be successful in its outcome as well as money saving. Although patients have to repeat ovum pick-up cycles and vitrify either oocytes or embryos, the PGD/PGS process and embryo transfer is performed only once. The revised manuscript has been corrected according to the referees' suggestions and I found it suitable now for publication. Although this is a retrospective study with heterogeneous patients but still small number of patients, the concept that raised by the author is worth presentation.

I have read this submission. I believe that I have an appropriate level of expertise to confirm that it is of an acceptable scientific standard.

# Peer review: Referee response for version 1

**DOI:** 10.5256/f1000research.2565.r3264

**Published:** 2014-01-30

**Authors:** Joep PM Geraedts

**Affiliations:** 1 Department of Genetics and Cell Biology, Maastricht University, Maastricht, Netherlands

The ability to accumulate oocytes or embryos from multiple cycles before PGD is done, could be an interesting development as is suggested in this article. However, the numbers are too small to be conclusive.

I agree with the remarks of the first referee, furthermore I would like to add the following points for revision:

Table 1:

a. The numbers of normal and abnormal embryos should be specified.

b. What are the definitions of clinical pregnancy rate per patient with transfer and the pregnancy rate per patient with transfer?

c. How many embryos were frozen?

d. How many FETs (Frozen Embryo Transfers) were done?

e. All percentages should be given using decimal points.

In the discussion it is stated that a high percentage of patients had an embryo transfer of healthy embryos. In my opinion this cannot be concluded from the material presented in the manuscript. In a number of cases FISH was used on day 3 embryos, which means that not PGS, not PGD was done. This screening can only give the results for the chromosomes included in the FISH analysis, while all other chromosomes can still be aneuploid. Furthermore it is known that mosaicism can complicate analysis at day 3.

Finally the information given with respect to the costs is not detailed enough. The prices of an ICSI cycle and the PGD analysis should be given in order to come to conclusions about the financial aspects of this approach.

I have read this submission. I believe that I have an appropriate level of expertise to confirm that it is of an acceptable scientific standard, however I have significant reservations, as outlined above.

# Response

**Published:** 2014-02-10

**Authors:** Martine Nijs

**Affiliations:** The Geertgen Foundation, Netherlands

We thank Prof. Dr. Geraedts for his questions, comments and suggestions on our manuscript. We have listed our replies below. They will be implemented in in the final version of the manuscript.

a. In total 40 embryos were diagnosed as normal, 43 as abnormal and for 9 embryos no result was obtained.

b. Clinical pregnancy is defined as the presence of a gestational sac with foetal heartbeat by ultrasound at 8-10 weeks after embryo transfer.

c. Eleven supernumerary embryos, diagnosed as being normal, were frozen post-embryo transfer.

d. No Frozen embryo transfer was done for these patients.

e. All percentages will be listed using decimal points.

The discussion will be rephrased and will state the following:  ‘As a consequence, a high percentage of patients had transfer of an embryo diagnosed to be negative for the specific genetic test (92,3%).’

Our cost calculation of the treatments was based on the following:

- Traditional strategy involves repeat hormonal stimulation, repeat oocyte collection, repeat ICSI  and repeat genetic diagnosis tests, each step associated with low chance for embryo transfer because of low number of oocytes collected.
- Cost of the accumulation strategy includes repeat hormonal stimulation, repeat oocyte collection, repeat vitrification and storage, one warming cycle, one ICSI and one genetic diagnosis test with very good chance for an embryo transfer.

# Peer review: Referee response for version 1

**DOI:** 10.5256/f1000research.2565.r2412

**Published:** 2013-11-20

**Authors:** Pedro Barri

**Affiliations:** 1 Department of Obstetrics, Institut Universitari Dexeus of the Universitat Autònoma de Barcelona, Barcelona, Spain

This is an interesting paper that addresses an important issue, but the sample size is small (13 patients) and there is relevant information lacking. I believe that this article should have the following recommendations taken into consideration:

1. Provide information concerning patients’ baseline characteristics (as well as age, AFC and hormonal profile).
2. Could the authors specify how allocation was done? (Please explain “based on the outcome of a medical counselling session with the patient”.)
3. In Methods, Ovarian stimulation of patients, in the phrase “to obtain an accumulated minimum of 6 oocytes” the word “mature” should be added after oocytes.
4. How was the cut-off of 6 mature oocytes established? Taking into account the average rates of fertilization and development to D2 embryos, isn’t this cut-off too low? (It is true that with this cut-off the obtained results are good but the sample size is small…)
5. In Results, the phrase “until a sufficient number was stored (< 6 mature oocytes or < 5 embryos on day 2)” : the < should be a >.
6. One patient did not have any healthy embryos for transfer: could you please explain how many embryos were biopsied in this patient?
7. The total number of embryos available for PGD is given for each group, but could you provide the mean number of biopsied embryos ± SD per patient?
8. If multiple pregnancy rate is zero it should be specified better, if it is not zero the rate should be given.
9. I agree with the conclusion of the study but, in order to firmly state that vitrification for accumulation purposes in PGD cycles increases the chances of success, the next 2 points should be taken into account:

- Was the rate of development to day 3 embryos the same in fresh and vitrified and warmed cycles?
- It could be interesting to analyze the euploidy rate between embryos coming from fresh oocytes vs. vitrified + warmed oocytes; the same for fresh vs. vitrified + warmed embryos.

I have read this submission. I believe that I have an appropriate level of expertise to confirm that it is of an acceptable scientific standard, however I have significant reservations, as outlined above.

# Response

**Published:** 2013-12-09

**Authors:** Martine Nijs

**Affiliations:** The Geertgen Foundation, Netherlands

We would like to thank Dr. Pedro Barri for his useful comments concerning our study on the evaluation of the efficacy of a PGD program in low responder patients after repeated ovarian stimulation and accumulation of vitrified oocytes or embryos. Indeed our sample size was small, but still indicative for the usefulness of vitrification as a tool for poor responders in a PGD/PGS program. Hence we opted to describe our observations in a ‘short’ research article.

1. The following baseline characteristics will be included in the revised article: Mean Age: 35.2 years; Mean AFC:7; Mean BMI: 24.6; Mean D2 FSH: 7.43.
2. This retrospective cohort study was performed over a 30 month-period (2011–2013). Patients were counseled on both options (serial oocyte or embryo vitrification) with clear explanations on the pro and cons of each option. Patients selected themselves for serial oocyte or embryo vitrification.
3. ‘Mature’ will be added to the specific sentence.
4. Our study population consisted of patients with a low number of eggs retrieved and embryos produced. Our cut off was the minimum number that was possible to be obtained by this patient population. A higher cut off would require additional stimulation cycles which was not an option, as it required more repetitive cycles and a higher treatment cost.
5. The < will be changed to >. 
6. This patient had 2 embryos from her first cycle and decided to vitrify them in order to proceed to another stimulated cycle. The aim was to increase the number of the available embryos for biopsy, and hence increase the number of having at least one healthy embryo to transfer after the screening. The next stimulation cycle resulted in 3 fresh embryos. In total 5 embryos were biopsied (2 thawed and 3 fresh). None of the embryos tested was genetically healthy and the transfer was cancelled.
7. The mean number of biopsied embryos per patient: 7.2  (+SD: 2.1).
8. Out of the 9 pregnancies obtained, two twin pregnancies were noted; both patients had delivery of healthy babies.
9. According to our in house data there is no difference in development or implantation rate of fresh versus vitrified embryos, hereby confirming results of Rienzi *et al*. (2009) and Ku *et al*. (2012). This observation can be included in the discussion part. Unfortunately, we do not have in house data on the euploidy status of vitrified oocytes. Forman *et al.* (2012) however, did not observe an increase in aneuploidy rates after vitrification and warming of embryos.
